# Deciphering the mechanisms of developmental disorders: phenotype analysis of embryos from mutant mouse lines

**DOI:** 10.1093/nar/gkv1138

**Published:** 2015-10-30

**Authors:** Robert Wilson, Christina McGuire, Timothy Mohun, the DMDD project

**Affiliations:** The Francis Crick Institute Mill Hill Laboratory, The Ridgeway, Mill Hill, London NW7 1AA, UK

## Abstract

The Deciphering the Mechanisms of Developmental Disorders (DMDD) consortium is a research programme set up to identify genes in the mouse, which if mutated (or knocked-out) result in embryonic lethality when homozygous, and initiate the study of why disruption of their function has such profound effects on embryo development and survival. The project uses a combination of comprehensive high resolution 3D imaging and tissue histology to identify abnormalities in embryo and placental structures of embryonic lethal lines. The image data we have collected and the phenotypes scored are freely available through the project website (http://dmdd.org.uk). In this article we describe the web interface to the images that allows the embryo data to be viewed at full resolution in different planes, discuss how to search the database for a phenotype, and our approach to organising the data for an embryo and a mutant line so it is easy to comprehend and intuitive to navigate.

## INTRODUCTION

The DMDD consortium (1) is characterising embryonic and perinatal lethal lines that have been generated as part of the Wellcome Trust Sanger Institute Mouse Genetics Project (MGP) pipeline (2) with the aim of identifying animal models useful for investigating the basis of human developmental disorders. Approximately a third of all mouse strains that carry a null mutation show homozygous recessive embryonic or perinatal lethality, and among this group at least 60% show structural defects in one or more organ system that can be readily detected in histological sections by conventional microscopy. Screening embryos in this fashion for unambiguous morphological abnormalities provides a remarkably efficient way to identify genes important for diverse aspects of normal embryo development. These genes are of interest to both developmental biologist and clinicians as they are potential candidates for disease alleles responsible for human developmental disorders. Characterisation of the abnormalities in the mouse lines should help us understand the genetic basis of human congenital disorders and potentially offer opportunities for developing novel therapies based on mouse models.

The embryonic or perinatal lethal lines studied by the DMDD project are triaged according to the stage of development at which lethality occurs in embryos homozygous for the mutant allele. Initially, we attempt to collect embryos from a lethal line at embryonic day 14.5 (E14.5), since by that stage the major period of organogenesis is complete. Embryos that survive to this stage of development are imaged comprehensively by high resolution episcopic microscopy (HREM) (3). Images are captured with a spatial resolution of 2–3 μm and can reveal changes in morphology that are impossible to detect with other techniques such as optical projection tomography (OPT), microcomputed tomography (μCT) or magnetic resonance imaging (MRI). When a line is not viable at E14.5, we attempt to obtain embryos at mid-gestation (E9.5). We also undertake whole embryo transcriptome analysis of carefully staged embryos at this early stage of development since this can offer a comprehensive molecular view of the downstream consequences of gene deletion. In addition, for the peri- and post-natal lethal lines that appear morphologically normal at E14.5, we carry out an observational screen for behavioural abnormalities (such as movement, responsiveness and suckling behaviour) shortly before birth (E18.5). Consecutive sections through the brain and spinal cord of embryos harvested at this late stage are stained with a set of established antibodies raised against neuronal antigens to reveal neuronal architecture. This relatively small category of lethals could help identify the genes and molecular mechanisms driving late phases of nervous system development and is important since defects in neuronal connectivity can give rise to a wide range of neurodevelopment disorders. In parallel with these studies we capture images of hematoxylin and eosin stained sections from the placenta of embryos from the lines to determine whether any of the embryo phenotypes we observe could be due to placental abnormalities. This is the first time that this aspect of development has been assessed systematically, and our observations will be described in a separate publication.

The images collected are screened systematically for morphological defects by a team of developmental biologists and anatomists. The protocol used to review the HREM data has been optimised allowing us to assess an embryo for abnormalities within a couple of hours (4). One of the issues encountered in screening embryos for phenotypes is that morphological change occurs rapidly in three dimensions during development, and within one litter the embryonic stage of littermates can vary considerably. To address this we have imaged a large number of normal mouse embryos to provide a reference set of images documenting wild-type development between stages E9.5 and E15.5. This resource complements the standard anatomical texts, such as the Atlas of Mouse Development (5), which has recently become available online (6), and has helped us review mutant embryos for morphological abnormalities. We record the mutant phenotypes observed by using terms from the Mammalian Phenotype Ontology (7), or our own controlled vocabulary that enables us to document features such as abnormal topology not yet represented in the ontology. All images from the embryos and placenta we have studied can be viewed and searched through the DMDD project website along with the phenotypes we have observed. As the project develops, we will also integrate this with neural data and whole embryo transcriptome analysis.

## OVERVIEW OF THE RESOURCE AND DATABASE CONTENT

**Figure 1.**
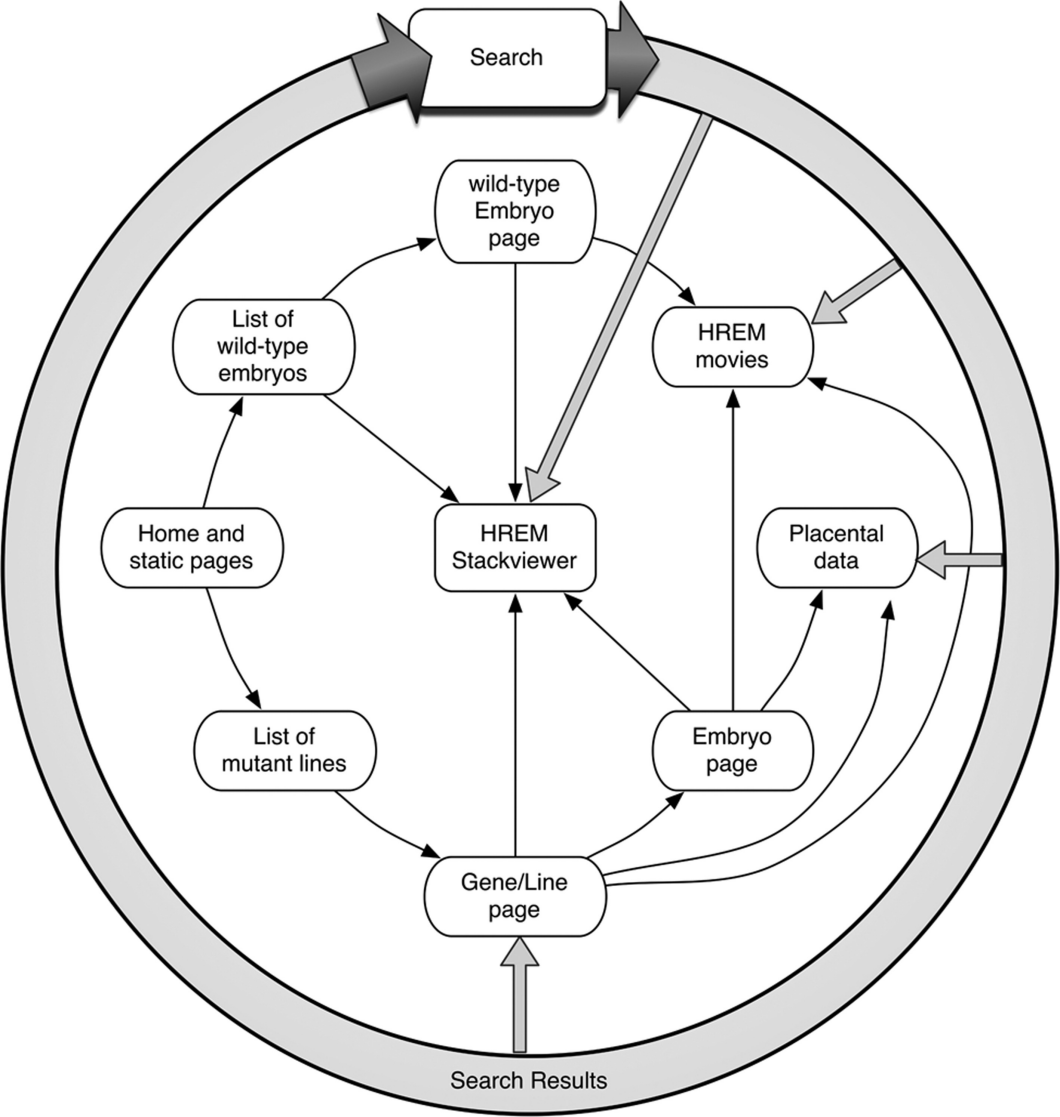
A schematic overview of the DMDD resource. The diagram shows how a user can navigate from the home page or one of the other static pages of the resource to view information about a gene, embryo or placenta, and how they are linked to the image data and movies associated with them. Users can browse the resource like this, however there is also a search function that offers access to the information about a specific gene, or the image data that characterises a particular phenotype that has been observed.

The organisation of the DMDD resource is shown in Figure 1. This illustrates that the resource can be used to view the data at the level of a gene or an embryo, and as outlined below, phenotype summaries are available for both of these perspectives. In addition, the search function allows users to find a specific gene and provides access to all the data collected across the resource relating to a particular phenotype. The resource is specifically designed to allow easy access to the images that are the basis of the phenotype statements.

The 2015.08 release of the resource uses a MySQL database to store information about 104 genes; there is morphological data on 495 embryos from 85 lines, and information about the placenta of 307 embryos from 45 lines. We have also collected HREM images of 110 wild-type embryos to use as a reference dataset. All placenta data has been scored for abnormalities and embryos from 25 lines have so far been reviewed for mutant phenotypes. A substantial number of embryos were imaged as part of a pilot HREM project before DMDD phenotyping was established and currently lack annotation. However, even in its absence, these images represent a useful resource and will be reviewed for mutant phenotypes systematically in the future. Due to the workflow of the project it is also possible that the placentas of some lines can be analysed before HREM images of the embryos have been collected and scored for phenotypes. The website currently contains 4.2 million HREM images recording embryo morphology and we expect to accumulate approximately one million HREM images a year obtained from annual analysis of around 50 lethal lines. Below we discuss how this large collection of image data can be explored through the *stackviewer* interface of the website, which can be accessed from an embryo or a gene report page, or from the results of a phenotype search. We will describe how the images in the *stackviewer* are tightly integrated with phenotype observations.

## DATA VISUALISATION

### The stackviewer

**Figure 2.**
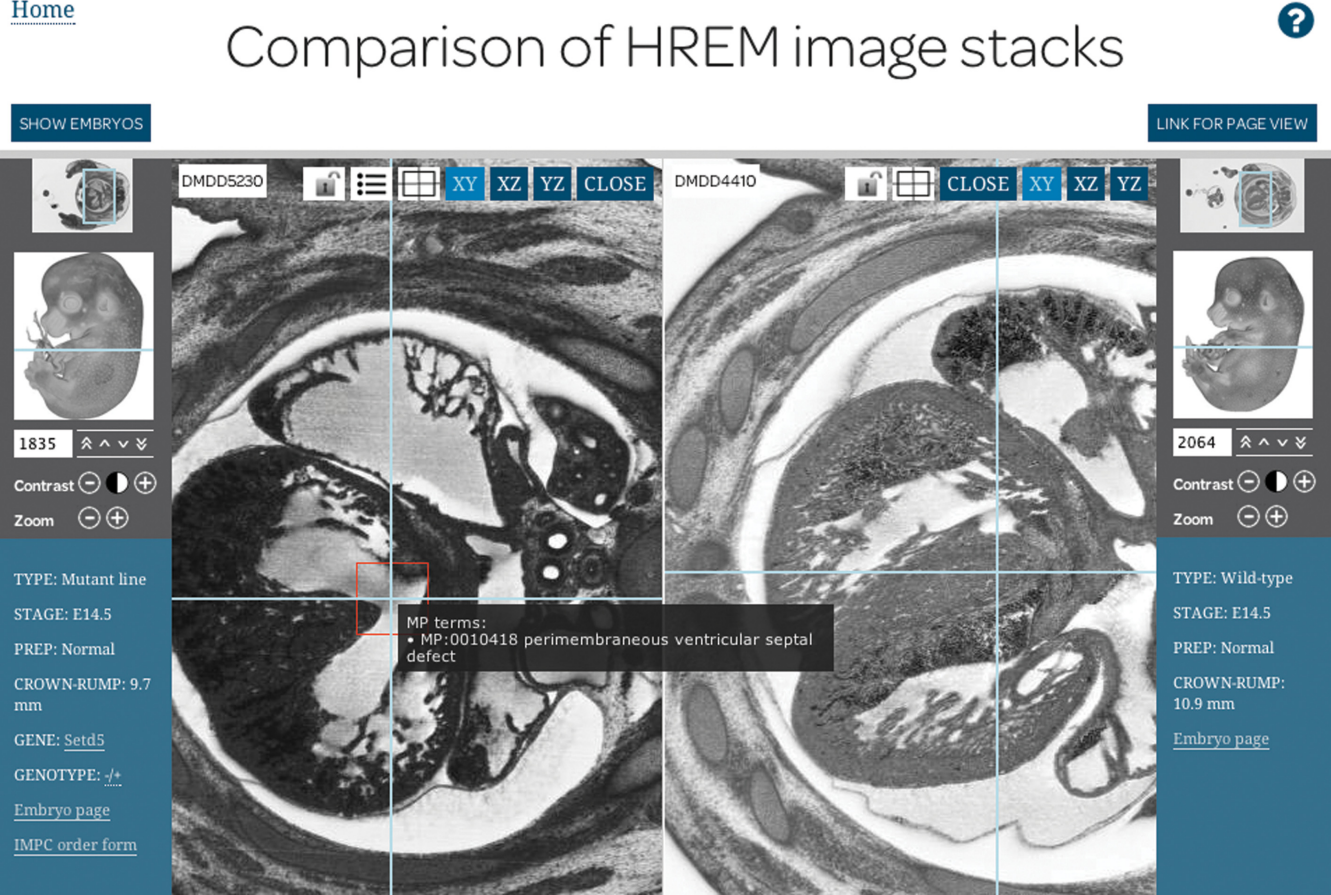
Comparison of two embryos in the *stackviewer* interface. To make it easy to navigate, each image displayed in the *stackviewer* has an overview window that indicates position within the image when viewed at high resolution, and there is also a 3D model of the embryo with a line indicating the position of the image within the HREM data stack. Dragging the line to a different position on the 3D model selects a section through that part of the embryo. For finer control the arrow keys can be used to move through the image stack by a single section, or in steps of 10 sections at a time. The number of the current section being viewed within the image stack is shown in the box of the stack navigation panel, and it is possible to move directly to a particular section by entering the number of the section in the box and pressing return. The main window of the *stackviewer* contains a set of light blue crosshairs. These are shown by default but can be turned on and off using the keyboard space bar. The crosshairs are a navigation tool indicating how the image data will be re-sliced when viewing another projection of the data, and can be repositioned with the mouse cursor. Images in the planes denoted by the crosshairs can be viewed by clicking on the ‘XY’, ‘XZ’ and ‘YZ’ buttons at the top right hand corner of the window. The icons adjacent to these buttons in the DMDD5230 window from right to left respectively: allow the crosshairs to be centred in the window, the mutant phenotypes scored for the embryo to be viewed, and enable the two windows to be locked together. Red frames highlight the locations of phenotype annotations in the main window, although these can be hidden by pressing the ‘a’ key on the keyboard. Hovering over a red frame reveals the phenotype scored at that location.

The morphology of the embryos recorded by HREM imaging can be reviewed in the *stackviewer* interface (Figure 2). There are a number of ways to display 3D data volumes over the web, such as Tissue Stack (https://github.com/NIF-au/TissueStack), IIP3D (8) and the WebGL framework, XTK (https://github.com/xtk/X#readme). However, for optimum performance we decided to develop an interface to accommodate 3D data sets from IIPMooViewer created by Ruven Pillay (http://iipimage.sourceforge.net/), which enables very large 2D images to be viewed over the web. The *stackviewer* works with a standard IIPImage server to provide fast access to HREM image data over the internet, allowing users to view an image at different magnifications in a similar fashion to the web interface of Google Maps, and select a specific location within the image stack. This makes it possible to review HREM data at the full resolution captured by the CCD camera. A particular view of a structure can be recorded by clicking the button labelled ‘LINK FOR PAGE VIEW’. This produces a URL describing the view (in similar fashion to the URLs generated by Google Maps), allowing any user-generated view to be replicated, easily documented or shared.

#### Linked orthogonal views

The raw HREM images are captured by imaging the block face as transverse sections are cut through the embryo, thus even though the serial set of aligned images describe the 3D volume comprehensively, the primary view of the data is limited to a single plane. The systematic phenotyping protocol we have developed to screen homozygous mutant embryos for morphological abnormalities is based upon examining the embryos in the axial, sagittal and coronal orthogonal planes. To generate these views we create a memory mapped 3D data structure from the HREM images using the Woolz library (8,9), and use this to produce a set of orthogonal projections through the data at full resolution for display over the web. These views of the data allow identification of a wide variety of structural abnormalities, some of which are more easily visualised in one particular plane. A set of movable crosshairs in the main window of the *stackviewer* makes it easy to navigate the image data (Figure 2). The crosshairs allow users to control how the image data will be re-sliced when the ‘XY’, ‘XZ’ and ‘YZ’ buttons in the top right hand corner of the window are used to view the data in another projection.

#### Annotation data

The images captured for homozygous mutants embryos are screened in a systematic fashion for phenotypes. When phenotype data exists for an embryo an icon appears at the top of the main *stackviewer* window, which can be seen in Figure 2 located immediately to the right of the padlock icon of the embryo DMDD5230. Clicking on this icon reveals a table listing all the phenotypes scored for the embryo. The phenotypes scored consist of standard terms from the Mammalian Phenotype Ontology (MP) and also some controlled vocabulary (CV) terms that are used by the DMDD project. These DMDD CV terms are either used to describe a phenotype when there is no suitable term within the Mammalian Phenotype Ontology, or as qualifiers, such as ‘normal for stage’, that are used in conjunction with a Mammalian Phenotype Ontology term or another DMDD CV term describing a phenotype. The qualifiers enable us to distinguish mutant phenotypes that are hard to call with certainty at the stage of analysis (for example a cleft palate at E14.5) from those that can be assigned unambiguously. We believe it is important that users can clearly see the basis of these phenotype assignments and clicking on a row in the phenotype table moves the *stackviewer* to the appropriate image, centred on the phenotype annotation. The features responsible for phenotype assignment are highlighted by a red frame which scales as image magnification is changed and is available in all three orthogonal views.

#### Comparison of image stacks

One of the challenges of having a large amount of image data online is to facilitate comparison of different datasets. The *stackviewer* interface has a panel at the top of the page, controlled by the ‘SHOW/HIDE EMBRYOS’ button, where the image data of any of the other embryos can be selected. Dragging one of the images of the embryos onto the main window creates a dual window display. To help orientate users, the second stack opens at the same relative position as the first. This enables direct comparison of two image stacks, allowing for example, the equivalent morphological structure to be viewed in both a wild-type and mutant embryo. Each stack can be manipulated independently by using its own stack controls. Once best matching views have been found, the windows can be locked together by clicking on the padlock icon. In this state, any action taken in one window is reflected in the other. In the dual window display mode, either dataset can be replaced by dragging another 3D model from the selection panel onto the appropriate stack window.

Another powerful way to explore the image data is to create a dual window display for a single embryo, by selecting the same embryo to view in each window. If the plane viewed is then changed in one of the windows and two windows linked together using the padlock icon, moving the crosshairs in one window updates the view in the other window. This allows structures to be dynamically traced through an embryo. This can be performed at the full resolution of the image data, something that would be difficult to achieve with such large datasets on a desktop computer.

### The embryo page

A panel adjacent to the main *stackviewer* window describes the embryo currently viewed, but more information is available on a dedicated page. This *embryo page* is divided into four sections. The ‘Info’ section contains links to the Mouse Genome Informatics (MGI) website (http://www.informatics.jax.org/) (10), allowing users to obtain more details on the gene and allele examined. This section also lists the genotype, crown-rump length, stage and background strain of the embryo studied, and provides a link to the International Mouse Phenotyping Consortium (IMPC) website (http://www.mousephenotype.org/) (11) where the colony can be ordered. The ‘HREM’ section contains a link to the *stackviewer* and also provides access to a set of small-scale movies that enable users to rapidly review the image data in all three orthogonal planes. An additional video produced by 3D reconstruction of the image data shows the external morphology of the embryo. All movies open in separate windows to allow multiple embryos to be compared. The phenotypes described for the embryo and its placenta are listed (when available) in the remaining two sections. The table of embryo phenotypes, like that accessed via the *stackviewer*, allows retrieval of image data underlying phenotype calls, clicking on any row opening the *stackviewer* at the appropriate image. The placenta phenotypes are also described by Mammalian Phenotype Ontology terms and each row of the placenta phenotypes section is similarly linked to the primary image data underpinning the phenotype statements.

### The gene/line page

**Figure 3.**
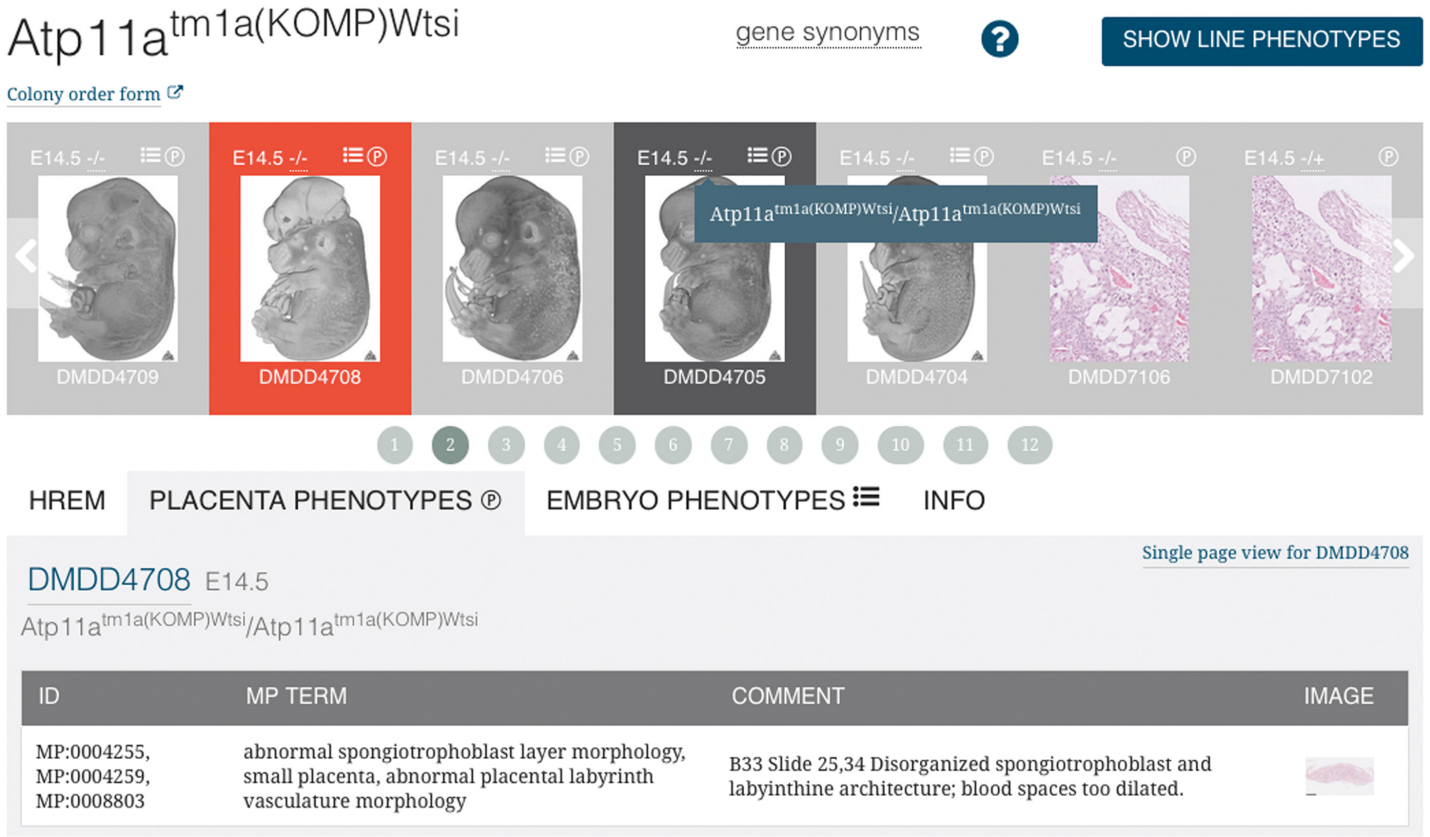
The gene page carousels. The top carousel shows images of the external morphology of the embryos studied for the allele, or a generic image of a placenta when that is the only kind of image data available. Each image is associated with the database id, stage and icon representing the genotype of the specimen. Hovering over the genotype icon reveals the genotype of the specimen. If phenotype annotations are available for the embryo or placenta these are indicated by the symbols found on the phenotype tabs of the bottom carousel. The embryo or placenta currently selected in the top carousel is highlighted in red, and the information and phenotypes described for this embryo are shown in the bottom carousel. The information is identical to that displayed on the embryo page; phenotype statements are linked to the original image data. The embryo selected can be changed by clicking on another embryo in the top carousel, using the arrows at the ends of the top carousel, or selecting one of the numbers below the carousel. The selection of another embryo updates the information in the bottom carousel. The tab viewed does not change as the update occurs allowing rapid screening of a subset of the data across the mutant line.

For every gene we have studied, the website has a *gene page* collating information about the embryos and placentas collected from all the lines carrying mutations that affect the gene. In most cases this will be a single allele but in a few cases, (for example with *Dbn1*) more than one allele has been examined and these are then listed consecutively on the *gene page*. Large amounts of data are collected for each allele and in order to keep the data compact and easy to review we have organised the information using two linked carousels (Figure 3). Tables that summarise the phenotypes of the homozygous mutant embryos and placentas can be displayed by clicking the ‘SHOW LINE PHENOTYPES’ button. These tables list the data according to penetrance of the phenotypes. By default the phenotypes identified are presented at the level of hierarchical detail used in the original scoring. However, it can also be useful to group together the phenotypes affecting a specific organ or process to obtain an overview of the principal tissues affected by the allele. This can be done using the INTERMEDIATE and HIGH LEVEL SUMMARY buttons, which dynamically re-map the designated Mammalian Phenotype Ontology terms, used to describe each phenotype, onto different ‘slims’ of the ontology to provide higher level views of the data.

## SEARCHING THE DATABASE

A list of all the genes studied can be found under the ‘MUTANTS’ item of the main menu bar. Icons within the table indicate the range of data types and annotations available. The gene list can be filtered by starting letter, and hovering over the letters above the table shows how many genes fall into each category. Clicking on a table row opens the *gene page*.

More complex queries can be performed using the search function, which allows searches based on either gene symbol or synonym, or observed phenotype. The search function is backed by a standalone SOLR server, because it is optimised for full-text searches rather than the storage and retrieval of records. The SOLR server is updated with information from the database during the preparation of each public release. The search function is available across the site via the ‘SEARCH’ button in the main menu bar. The *search pane* drops down over the main window: the two tabs in the pane enable users to choose the type of query. By default the *search pane* is set to run a phenotype search for a term from both the Mammalian Phenotype Ontology, and the DMDD controlled vocabulary. The *phenotype search* tab also allows users to search the free text comments that have been added to describe the phenotypes. An autocomplete function makes suggestions based on the ontology terms that have been used to record the mutant phenotypes. Users can query the database by selecting one of these suggestions, entering a Mammalian Phenotype Ontology term or id, or using a DMDD controlled term. By default, search results will include matches to the search term and to ‘children’ of the term within the ontology although this behaviour can be optionally modified. This offers the advantage of enabling searches using high level ontology terms to identify a broad range of associated phenotypes.

The results of a *phenotype search* are listed in a table that by default is organised according to the allele found. Results can also be sorted according to the contents of each column. Each row of the results table is linked to the image showing the basis of the phenotype statement. Clicking on the row opens the image in a new window that has a red frame highlighting the region where the phenotype is observed, and a link to the *stackviewer* allowing the phenotype to be examined in greater detail. An alternative way to review search results is the ‘*Thumbnail view*’. In this mode, thumbnail images of sections show the phenotypes identified by the search, and there are views of the external morphology of the embryos identified. Results are grouped according to the alleles identified in the search. The thumbnails provide links to the appropriate image data which open in separate windows.

The *gene search* tab performs a case-insensitive search for gene symbols and synonyms by default, although searches can be restricted to valid symbols. An autocomplete function suggests symbols and synonyms of genes studied in the DMDD project, which can be selected using the arrow keys or the mouse cursor. A single result returns the appropriate *gene page*, while several results produce a table listing the symbol, name and the synonyms of the genes where each row is linked to the corresponding *gene page*.

## DISCUSSION AND FUTURE WORK

The central challenge in constructing the web interface to the DMDD database has been to integrate the enormous quantity of images and assigned phenotypes in a structure that is intuitive to navigate, readily searchable, responsive and that facilitates detailed review of the data by users. Whilst the DMDD project focuses on imaging embryos from mutant mouse lines, similar challenges are increasingly faced throughout biomedical research. Novel imaging methodologies, automation methods and computational advances frequently now converge to produce previously unimaginable quantities of data that require new ways to be stored, maintained and mined for new biological insights. At the heart of the solution we have developed is the *stackviewer* page, which provides a very effective and flexible way to store and present terabyte quantities of image data whilst maintaining full image resolution. This approach could readily be applied to manage data from any research project that produces similarly large image sets and which are required to be readily available for review or comparison.

One important factor that dictates the scale of the data required by DMDD is the possibility, confirmed in our studies, that phenotypes produced by individual gene mutations can vary in their penetrance. As a result, it is necessary to analyse a group of mutant embryos, with the consequent issue of how best to present and summarise phenotype data from each cohort. Again, this is likely to be a common problem for large scale biomedical projects where genetics or stochastic mechanisms result in similar individual variability. For the DMDD website, we have addressed this problem by organising our data in an effective hierarchy of pages, from the *embryo page* that brings together all information from a single embryo and provides access to the *stackviewer*, to the *gene page* that presents the range of data obtained from all embryos of a particular mutant line. As yet, the approach we have taken to summarising phenotype data for each line is relatively simple, based on frequencies with which particular abnormalities are detected. An important future goal is to incorporate summaries (most likely graphical) that can reveal potential associations between different individual abnormalities, since this data will have particular value for investigating their mechanistic origins.

The goal of the DMDD consortium is to identify mouse null alleles that mimic aspects of human congenital disease in order to catalyse research into the developmental basis of such abnormalities. Our database and website is therefore designed to provide a resource for both developmental biologists and clinicians. An effective and flexible search tool is essential for success. The foundation for this is structured annotation of all data. We have used the Mammalian Phenotype (MP) Ontology as the basis for this and addressed its limitations for embryo analysis by creating a controlled vocabulary that is currently being incorporated into the MP ontology. The search tool itself allows either gene-based or phenotype-based searches but we appreciate that a developmental biologist may approach the information we capture in different ways from a clinician. We believe it is essential that this is reflected in the search tool and to achieve this we aim to incorporate searching via other ontologies where mapping of terms has been agreed. Future releases of the DMDD website will allow users to search with a mouse or human anatomy ontology term since these can be dynamically mapped onto the Mammalian Phenotype Ontology (and hence our data). Our goal is to extend this to encompass searches based explicitly on human disease syndromes. Addition of late gestation neural phenotypes will broaden the utility of the database; incorporating genome-wide expression profiles of the mutant and control wild-type littermates has the potential to directly link mouse mutant lines to the wealth of human genetic data emerging from disease genome sequencing studies.
